# Sodium hydrosulfide attenuates cerebral ischemia/reperfusion injury by suppressing overactivated autophagy in rats

**DOI:** 10.1002/2211-5463.12301

**Published:** 2017-09-21

**Authors:** Wen‐Wu Jiang, Bai‐Sheng Huang, Yang Han, Lv‐Hong Deng, Li‐Xiang Wu

**Affiliations:** ^1^ Department of Physiology Xiangya School of Medicine Central South University Changsha China; ^2^ Department of Neurosurgery the First Affiliated Hospital of University of South China Hengyang China; ^3^ The First Affiliated Hospital of University of South China Hengyang China

**Keywords:** autophagy, cerebral ischemia/reperfusion injury, hydrogen sulfide, oxygen–glucose deprivation/reoxygenation, rat, sodium hydrosulfide

## Abstract

Ischemic stroke is a leading cause of death and disability worldwide, and autophagy may be involved in the pathological process of cerebral ischemia/reperfusion injury. Hydrogen sulfide (H_2_S) is an endogenous gasotransmitter with protective effects against multiple diseases. Here, we tested the effect of H_2_S on cerebral ischemia/reperfusion injury in rats. Sodium hydrosulfide (NaHS), an H_2_S donor, improved neurological function and reduced the size of the infarcts induced by transient middle cerebral artery occlusion (MCAO) followed by reperfusion in rats. NaHS treatment reduced the lactate dehydrogenase (LDH) activity in the serum (a marker of cellular membrane integrity) and the expression of cleaved caspase‐3 (a marker for apoptosis) in the brains of MCAO rats. We also found that autophagy was overactivated in the brains of MCAO rats, as indicated by an increased ratio of LC3 II to I, decreased expression of p62, and transmission electron microscope detection. NaHS treatment significantly inhibited the autophagic activity in the brains of MCAO rats. Furthermore, PC12 cells were subjected to oxygen–glucose deprivation/reoxygenation (OGD/R) to mimic MCAO *in vitro*. We found that NaHS treatment reduced cellular injury and suppressed overactivated autophagy induced by OGD/R in PC12 cells. An autophagy stimulator (rapamycin) eliminated the protective effect of NaHS against LDH release and caspase‐3 activity induced by OGD/R in PC12 cells. An autophagy inhibitor (3‐methyladenine, 3‐MA) also reduced the cellular injury induced by OGD/R in PC12 cells. In conclusion, the results indicate that overactivated autophagy accelerates cellular injury after MCAO in rats and that exogenous H_2_S attenuates cerebral ischemia/reperfusion injury via suppressing overactivated autophagy in rats.

**Abbreviations**
3‐MA: 3‐methyladenine
CBS: cystathionine β‐synthase
CSE: cystathionine γ‐lyase
H_2_S: hydrogen sulfide
I/R: ischemia/reperfusion
LDH: lactate dehydrogenase
MCAO: middle cerebral artery occlusion
NaHS: sodium hydrosulfide
OGD/R: oxygen–glucose deprivation/reoxygenation
TEM: transmission electron microscope

Cerebral infarction is a main cause of death and permanent disability in adults worldwide 1. Cerebral ischemia‐induced brain cell damage is further aggravated when the blood supply is restored, and this is referred to as ischemia/reperfusion (I/R) injury 2. Recently, many studies have focused on cerebral ischemia/reperfusion injury. However, the exact mechanism of cerebral ischemia/reperfusion injury is still not completely understood, and effective therapeutic methods for the treatment of ischemic stroke are needed.

Autophagy is a highly regulated process involving the bulk degradation of cytoplasmic macromolecules and organelles in mammalian cells 3. Autophagy is involved in many pathophysiological situations, such as inflammation 4, apoptosis 5, malignancy 6, 7, and cerebral ischemia/reperfusion injury 8. However, the exact role of autophagy in cerebral ischemia/reperfusion injury remains controversial. Su *et al*. 9 report that autophagy activation contributes to the neuroprotective effect of remote ischemic preconditioning against focal cerebral ischemia in rats. Fan *et al*. 10 also found that ischemic preconditioning enhances autophagy but suppresses autophagic cell death in rat spinal neurons following ischemia/reperfusion, while other reports have found that inhibition of autophagy is protective against ischemia/reperfusion injury. An NMDA glutamate receptor antagonist, Ro25‐6981, suppresses ischemic brain injury by inhibiting autophagy 3. Inhibition of the mitochondrial calcium uniporter also protects neurocytes against ischemia/reperfusion injury by downregulating excessive mitophagy 11. Further investigating the role of autophagy in cerebral ischemia/reperfusion injury is an important priority. In this study, we propose that overactivated autophagy contributes to cerebral ischemia/reperfusion injury.

Hydrogen sulfide (H_2_S) is the third endogenous gasotransmitter to be discovered, following nitric oxide (NO) and carbon monoxide (CO) 12. H_2_S is mainly produced from L‐cysteine by cystathionine β‐synthase (CBS) and cystathionine γ‐lyase (CSE) in mammalian tissue 13. H_2_S influences cellular and organ functions by a number of different mechanisms 14. It is well known that H_2_S protects nerves against oxidative stress 15, defends the kidneys from ischemia/reperfusion injury 16, and regulates endoplasmic reticulum stress 17. Accumulated evidence indicates that H_2_S exerts a protective effect against ischemia/reperfusion injury, such as myocardial ischemia/reperfusion injury 18 and kidney ischemia/reperfusion injury 16. Recently, it has been reported that H_2_S attenuates cerebral ischemia/reperfusion injury in a mouse model 19. Nonetheless, the exact mechanisms and identities of molecules responsible for the neuroprotective effect of H_2_S against cerebral ischemia/reperfusion injury remain incompletely defined.

In this study, we propose that overactivated autophagy contributes to cerebral ischemia/reperfusion injury and that H_2_S attenuates cerebral ischemia/reperfusion injury by inhibiting autophagy. Therefore, we investigated the alteration of autophagy in the brain after ischemia/reperfusion in rats. Sodium hydrosulfide (NaHS), a donor of H_2_S, was used to test the effect of H_2_S on cerebral ischemia/reperfusion and autophagy in a well‐established rat model.

## Materials and methods

### Ethics statement

All experiments were approved by the Institutional Animal Care and Use Committee (IACUC) at the Central South University. No distress vocalization, prostration, or hyperactivity was observed throughout the experiment.

### Experimental animals

Forty adult male Sprague‐Dawley rats, weighing between 250 and 270 g, were singly housed in smooth‐bottomed plastic cages in a colony room maintained on a 12‐h light/dark cycle. The animals were allowed free access to rodent chow and water. To accustom the animals to the laboratory environment, an acclimation period of 1 week was allowed before the initiation of the experiment. All rats were grouped into the control group, the middle cerebral artery occlusion (MCAO) group, and the MCAO+NaHS group. The surgical procedure of MCAO was described below. The concentration of NaHS used in this study was 5.6 mg/kg according to the previous research 20.

### Surgical procedure: cerebral ischemia induced by MCAO

Briefly, as described in a previous study 21, anesthesia was induced with 5% isoflurane and maintained with 2.5% isoflurane through a facemask. The rectal temperature was monitored and kept at 37 ± 0.5 °C using a feedback‐regulated heating system during surgery, supplemented with oxygen. A 0.2 mL injection of 0.5% bupivacaine was given subcutaneously at the prospective incision site. Permanent focal ischemia was induced by intraluminally occluding the right middle cerebral artery (MCA). A 4‐0 nylon monofilament suture (total length: 3 cm; silicon‐coated tip length and diameter: 5 mm and 0.39 ± 0.02 mm, respectively; MCAO suture PK10, 40‐333PK10, Redland, CA, USA) with a slightly enlarged round tip was inserted into the stump of the external carotid artery (ECA) and pushed approximately 18‐20 mm from the carotid bifurcation. Blood flow was thus blocked at the MCA origin. The monofilament was carefully removed 2 h later to restore blood flow, and the tissue was reperfused for 24 h. Finally, the skin was sutured and the animals were returned to their cages. Sham‐operated animals were subjected to the procedures described above, except for suture insertion.

### Evaluation of neurological functional score

Neurological functional scores were evaluated at 24 h after reperfusion in six rats from each group, randomly chosen by staff blinded to the group identities. The test consists of two aspects of neurological function, as previously described 22: (a) the postural reflex test to examine upper body posture while the animal is suspended by the tail and (b) the forelimb placing test to examine sensorimotor integration in forelimb placing responses to visual, tactile, and proprioceptive stimuli. Neurological function was graded on a scale of 0 to 12 (normal score, 0; maximal score, 12).

### Infarct size

Twenty‐four hours after reperfusion, eight rats from each group were chosen randomly by staff members. The rats were decapitated under deep anesthesia, and the brains were rapidly removed. The infarct sizes were measured by staining with 1% 2,3,5‐triphenyltetrazolium chloride (TTC; Sigma‐Aldrich, St. Louis, MO, USA). The brains were cut into 2‐mm‐thick coronal sections in a cutting block. Then, the slices were stained with 1% TTC solution for 30 min at 37 °C followed by overnight immersion in 4% paraformaldehyde. The infarct area was measured in each slice with NIH image analysis software (imagej, Bethesda, MD, USA). As described previously 23, the size of the brain infarct as a percentage was computed by normalizing to the entire brain.

### Cell cultures

PC12 cells (Culture Collection of the Chinese Academy of Science, Shanghai, China) were cultured with RPMI‐1640 (Gibco, Carlsbad, CA, USA) containing 10% heat‐inactivated fetal bovine serum (Gibco). The cells were incubated under an atmosphere of 95% air and 5% CO_2_ at 37 °C.

### Oxygen–glucose deprivation/reoxygenation

As described previously 24, PC12 cells were washed once with phosphate‐buffered saline (PBS) and incubated in RPMI‐1640 containing no glucose or serum. Subsequently, the cells were incubated in a hypoxia chamber (Thermo Scientific, Waltham, MA, USA) with nitrogen (95% N_2_, 5% CO_2_) in a 60% humidified airtight chamber for 5 min. This process of oxygen–glucose deprivation/reoxygenation (OGD/R) routinely achieved O_2_ levels between 0% and 1% within the chamber. The cells were incubated within the chamber for an additional 4.5 h at 37 °C. After hypoxia, the cells were transferred back to full culture media with oxygen for 24 h. As a control, normal cells were incubated in a regular cell culture incubator under normal conditions.

### Transmission electron microscope

The brain tissue and PC12 cells were fixed with 2% paraformaldehyde/2% glutaraldehyde/0.5% CaCl_2_ (pH 7.4) for more than 6 h. After being washed with 0.1 m phosphate buffer, the samples were fixed in 1% OsO_4_ for 2 h. Then, the samples were dehydrated and embedded in araldite. Portions of the embedded tissue containing suitable cells were selected by light microscopy, cut out, and affixed to a small metal dowel. The tissue was sectioned in a Porter‐Blum microtome with glass knives, mounted on bare grids, stained with 1.0% KMnO_4_, and observed with a JEM‐1011 JEOL transmission electron microscope.

### Lactate dehydrogenase release assays

Cellular membrane integrity was monitored with a permeability assay based on the release of lactate dehydrogenase (LDH) into the media. Briefly, serum of rats or the supernatants of PC12 cells after treatment were transferred to a new 96‐well plate for LDH activity analysis. Next, 100 μL of LDH reaction solution was added to each well, the plate was incubated at room temperature for 30 min, and the absorbance was read at 490 nm using a Varioskan Flash 3001 microplate reader (Thermo Fisher Scientific). The intensity of the red color represented the LDH activity in the serum or supernatant.

### Flow cytometric detection of apoptosis

PC12 cells were plated in six‐well plates (Costar, New York, NY, USA) in duplicate with 1 × 10^5^ cells per well in 1 mL media, then incubated for 24 h. After OGD/R treatment, the media were removed. The cells were collected after trypsinization with 1× trypsin/EDTA, and then they were placed in 15‐mL tubes and centrifuged (600 ***g*** for 3 min). The media were removed, and the cells were washed twice with sterilized PBS and suspended in 1× binding buffer at a concentration of 1 × 10^6^ cells·mL^−1^. One hundred microliters of the solution was transferred to a fresh tube, and then 5 μL of FITC annexin V (BD Biosciences, Franklin Lakes, NJ, USA) and 5 μL of propidium iodide (BD Biosciences) at 10 μg·mL^−1^ final concentration were added to each tube. All cells were incubated for 15 min at room temperature in the dark, and the cell distribution was analyzed using a FACScan Flow Cytometer (Becton Dickinson, North Ryde, Australia) and flowjo analysis software (Ashland, OR, USA).

### Caspase‐3 fluorescence assay

PC12 cells (1 × 10^4^ cells per well) were seeded into sterile white (opaque) 96‐well plates (Costar). After treatment, a study of caspase‐3 activity was performed in triplicate using Caspase‐Glo 3 assay kits (Promega, Madison, WI, USA) according to the manufacturer's protocol. Briefly, 100 μL of Caspase‐Glo reagent was added and incubated at room temperature for 30 min. Activated caspases‐3/7 cleaved the aminoluciferin‐labeled synthetic tetrapeptide, releasing the luciferase substrate. Caspase‐3 activity was measured using a Varioskan Flash 3001 microplate reader (Thermo Fisher Scientific).

### Protein extraction and western blot analysis

PC12 cells were washed twice with cold PBS, scraped on ice, and centrifuged at 5000 ***g*** for 5 min. The resulting pellet was then sonicated in lysis buffer (62.5 mm Tris/HCl, pH 6.8 at 25 °C), 2% w/v SDS, 10% v/v glycerol) with a protease inhibitor cocktail. Immediately after being harvested, whole‐cell lysates were boiled for 10 min. Fifty micrograms of total protein was electrophoresed on SDS/12% polyacrylamide gels and transferred to a PVDF membrane (Millipore, Billerica, MA, USA). The membranes were blocked with 5% nonfat milk at room temperature for 2 h. Afterward, the membranes were incubated with primary antibodies against LC3 (1:500, Cell Signaling Technologies, Boston, MA, USA), p62, caspase‐3, or β‐actin as loading control (1:1000, Santa Cruz Biotechnology, Dallas, TX, USA) overnight at 4 °C. The PVDF membranes were then washed three times for 10 min at room temperature and incubated for 1 h with a horseradish peroxidase (HRP)‐linked anti‐rabbit IgG secondary antibody (1:5000) (Cell Signaling). After three‐time washes, the membranes were developed using the ECL Prime Western Blotting Detection reagent as specified by the manufacturer (Amersham Pharmacia, Buckinghamshire, UK). Band intensities were analyzed using imaging software (Bio‐Rad, Hercules, CA, USA), and the results were normalized to the β‐actin loading control.

### Statistical analysis

The results were expressed as the mean ± SEM. Statistical analysis was performed using spss 15.0 (Chicago, IL, USA). Intergroup differences were tested with ANOVA. For all tests, a *P* < 0.05 was considered significant.

## Results

### NaHS attenuated cerebral ischemia induced by MCAO in rats

**Figure 1:**
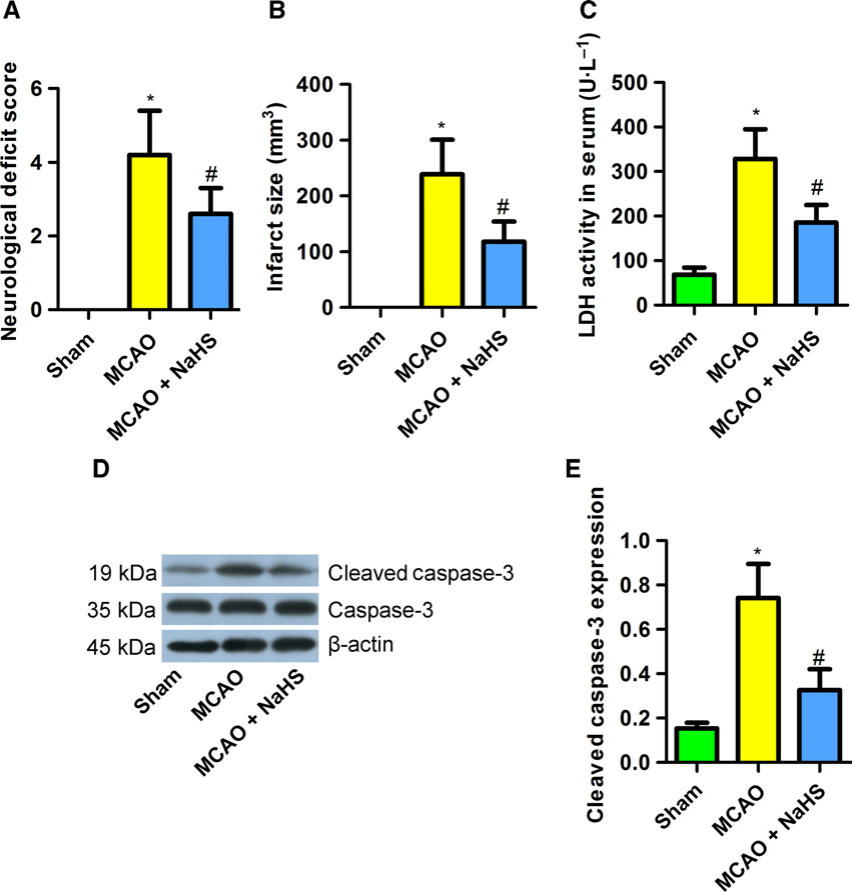
Sodium hydrosulfide attenuated cerebral ischemia induced by MCAO in rats. (A) NaHS reduced the neurological deficit score in MCAO rats (*n* = 6). (B) NaHS reduced the infarct size in MCAO rats (*n* = 8). (C) NaHS decreased the LDH activity in the serum of MCAO rats (*n* = 8). (D,E): NaHS reduced the expression of cleaved caspase‐3 in MCAO rats (*n* = 4‐6). *P < 0.05 compared with the sham group; #*P* < 0.05 compared with the MCAO group.

To determine whether NaHS supplementation could protect neurons against ischemic damage, we examined neurological deficits and infarct volume in MCAO rats. The results showed that NaHS (5.6 mg·kg^−1^) supplementation significantly improved neurological function (Fig. 1A) and reduced infarct size (Fig. 1B). NaHS also reduced the LDH activity in the serum of MCAO rats (Fig. 1C) and the protein expression of cleaved caspase‐3 in the brains of MCAO rats (Fig. 1D,E). These results indicate that NaHS attenuates cerebral ischemia induced by MCAO in rats.

### NaHS inhibited autophagy in the brains of MCAO rats

**Figure 2:**
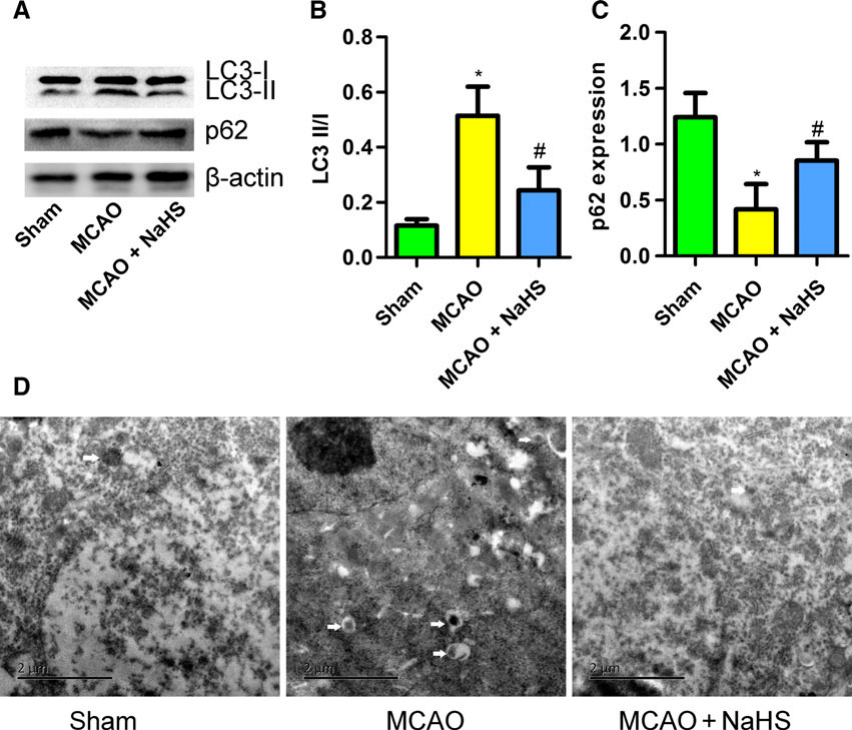
Sodium hydrosulfide inhibited autophagy in the brains of MCAO rats. (A) The expression of LC3 II/I and p62, detected by western blotting. (B) NaHS decreased the ratio of LC3 II to I in the brains of MCAO rats (*n* = 6). (C) NaHS increased the expression of p62 in the brains of MCAO rats (*n* = 6). (D) Transmission electron microscope images of brain sections (white arrow: autophagolysosome). *P < 0.05 compared with the sham group; #*P* < 0.05 compared with the MCAO group.

Then, we investigated the activation of autophagy in the brains of MCAO rats. MCAO increased the ratio of LC3 II to I and decreased the protein expression of p62 (Fig. 2A–C), indicating an increase in autophagy in MCAO rats. NaHS supplementation decreased the ratio of LC3 II to I but increased p62 expression (Fig. 2A–C). In addition, the transmission electron microscope (TEM) images showed that NaHS decreased the number of autophagolysosomes in MCAO‐treated rat brains. These results indicate that NaHS inhibits overactivated autophagy in the brains of MCAO rats, contributing to the protective effect of this compound against cerebral ischemia.

### NaHS reduced the cellular injury induced by OGD/R in PC12 cells

**Figure 3:**
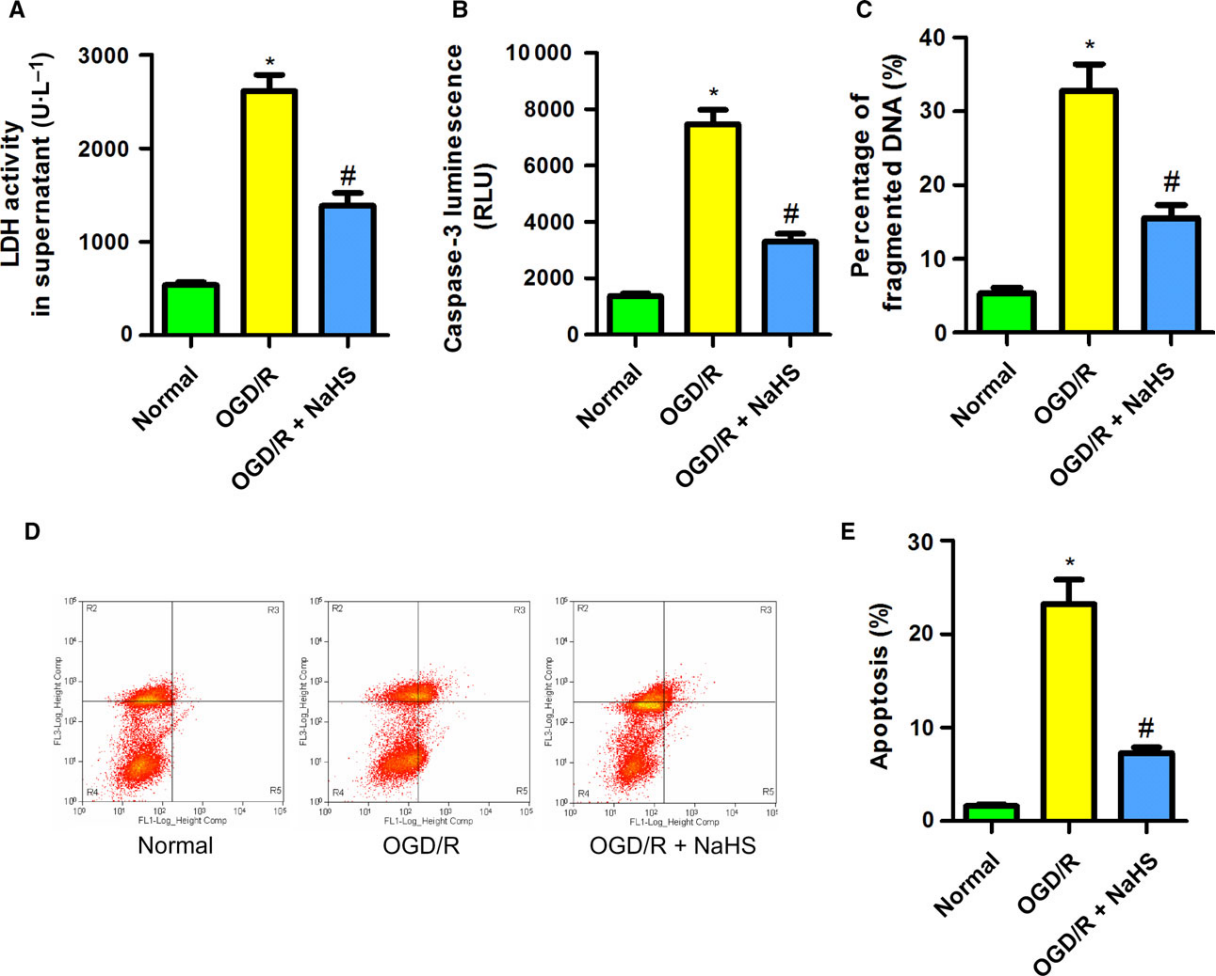
Sodium hydrosulfide reduced the cellular injury induced by OGD/R in PC12 cells. (A) NaHS reduced the LDH activity induced by OGD/R in the supernatant of PC12 cells (*n* = 6). (B) NaHS reduced the caspase‐3 luminescence induced by OGD/R in PC12 cells (*n* = 8). (C) NaHS decreased the percentage of fragmented DNA induced by OGD/R in PC12 cells (*n* = 6). (D,E) NaHS reduced the apoptosis induced by OGD/R in PC12 cells, as detected by flow cytometry (*n* = 6). *P < 0.05 compared with the normal group; #*P* < 0.05 compared with the OGD/R group.

To confirm the protective effect of NaHS against MCAO rats, PC12 cells were subjected to OGD/R, which was used to mimic MCAO *in vitro*, with or without NaHS (100 μm). The LDH activity in the supernatant was monitored to reflect cellular membrane integrity. We found that OGD/R increased the LDH activity in the supernatant of PC12 cells, which was attenuated by NaHS (Fig. 3A). This indicated that H_2_S protected PC12 cells against OGD/R‐induced cellular injury. Apoptosis plays a vital role in cell fate under stress. Active caspase‐3 and fragmented DNA are the markers for cells undergoing apoptosis. Interestingly, NaHS treatment significantly reduced the caspase‐3 activity and the percentage of fragmented DNA in PC12 cells subjected to OGD/R (Fig. 3B,C). We also assessed cell apoptosis using flow cytometry with FITC annexin V and propidium iodide staining. These results showed that OGD/R increased the percentage of apoptotic cells of PC12 cells. NaHS treatment attenuated the percentage of apoptosis (Fig. 3D,E). These findings suggest that NaHS has a protective effect against cellular injury induced by OGD/R in PC12 cells.

### NaHS suppressed autophagy induced by OGD/R in PC12 cells

**Figure 4:**
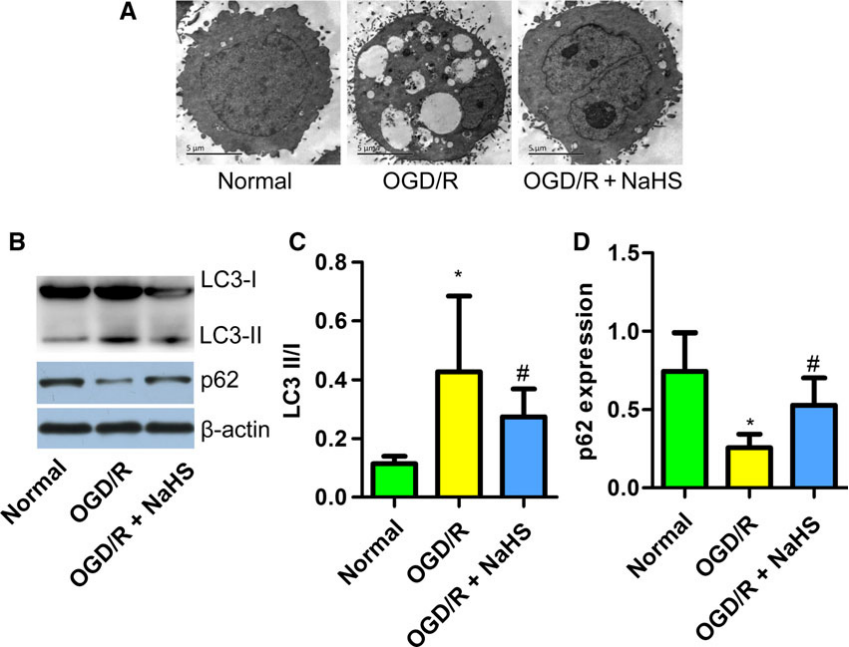
Sodium hydrosulfide suppressed autophagy induced by OGD/R in PC12 cells. (A) Transmission electron microscope was used to detect autophagolysosomes in PC12 cells. (B) A western blot detecting LC3 and p62. (C) NaHS reduced the LC3 II/I ratio in OGD/R‐treated PC12 cells (*n* = 6). (D) NaHS increased the expression of p62 in OGD/R‐treated PC12 cells (*n* = 6). *P < 0.05 compared with the normal group; #*P* < 0.05 compared with the OGD/R group.

We sought to clarify whether NaHS attenuated the PC12 cellular injury induced by OGD/R by suppressing autophagy. First, TEM was employed, and the results showed that NaHS treatment reduced the number of autophagolysosomes (Fig. 4A). Then, we detected the expression of LC3 and p62 by western blotting. We found that OGD/R treatment increased the ratio of LC3‐II to I and decreased the expression of p62, indicating an increase in autophagy in PC12 cells. NaHS treatment partly reversed the change in the LC3‐II/I ratio and restored the expression of p62 to a normal level (Fig. 4B–D). These results suggest that NaHS suppresses autophagy induced by OGD/R in PC12 cells.

### NaHS attenuated the PC12 cellular injury induced by OGD/R by suppressing autophagy

**Figure 5:**
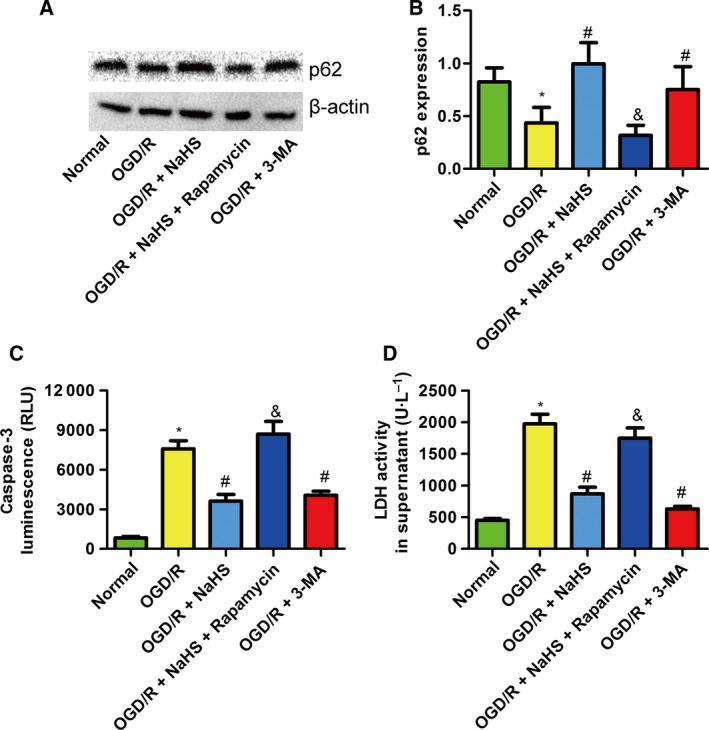
Sodium hydrosulfide attenuated the PC12 cellular injury induced by OGD/R by suppressing autophagy. (A) A western blot detecting p62 in PC12 cells. (B) Rapamycin, a stimulator of autophagy, decreased the expression of p62, but 3‐MA, an inhibitor of autophagy, increased the expression of p62 (*n* = 4). (C) Rapamycin increased the caspase‐3 luminescence compared with NaHS treatment, but 3‐MA decreased the caspase‐3 luminescence compared with OGD/R treatment (*n* = 4). (D) Rapamycin increased the LDH activity in the supernatant compared with NaHS treatment, but 3‐MA decreased the LDH activity in the supernatant compared with OGD/R treatment (*n* = 4). *P < 0.05 compared with the normal group; #*P* < 0.05 compared with the OGD/R group; &*P* < 0.05 compared with the OGD/R + NaHS group.

An autophagy stimulator (rapamycin) and inhibitor (3‐methyladenine, known as 3‐MA) were used. Western blot results showed that rapamycin decreased the expression of p62, and 3‐MA increased this expression (Fig. 5A,B). In addition, rapamycin eliminated the protective effect of NaHS against caspase‐3 activity (Fig. 5C) and LDH release (Fig. 5D) induced by OGD/R in PC12 cells. Interestingly, 3‐MA treatment without NaHS also reduced the supernatant LDH activity and caspase‐3 activity of PC12 cells (Fig. 5C,D). These results indicate that overactivated autophagy contributes to cellular injury caused by OGD/R in PC12 cells and that NaHS attenuates the cellular injury partly by suppressing overactivated autophagy.

## Discussion

Although the effect of H_2_S on cerebral ischemia/reperfusion injury has attracted the interest of researchers, the exact underlying mechanism remains unclear. Therefore, the hypothesis of this study is that H_2_S protects the brain against ischemia/reperfusion injury through an autophagy‐associated pathway. In the present study, our data demonstrated that autophagy was overactivated in rat brains subjected to ischemia/reperfusion injury. Exogenous supplementation of NaHS attenuated MCAO‐induced cerebral ischemia/reperfusion injury in rats and OGD/R‐induced cellular injury in PC12 cells, partially by inhibiting overactivated autophagy.

Autophagy is a highly regulated process that is involved in many pathophysiological situations, including cerebral ischemia/reperfusion injury 8, 25. Autophagy has been considered a double‐edged sword with prosurvival or prodeath potential in cerebral ischemia/reperfusion injury 26. Although ischemic preconditioning‐induced autophagy contributes to neuroprotection against ischemia/reperfusion injury 9, 10, the data in this study indicate that autophagy is overactivated in rat brains subjected to MCAO and PC12 cells subjected to OGD/R and that inhibition of autophagy by NaHS attenuates ischemia/reperfusion injury both *in vivo* and *in vitro*. Mounting evidence indicates that cerebral ischemia/reperfusion injury induces autophagy‐like cell death, also called type II programmed cell death 27. For example, NMDA receptor antagonists suppress brain ischemic injury by inhibiting autophagy, and rapamycin, a stimulator of autophagy, aggravates cerebral ischemia/reperfusion injury 3. In addition, inhibition of autophagy by 3‐MA prevents neuronal injury 28. In consideration of these reports and our findings, we believe that overactivated autophagy is a crucial factor for cell injury during cerebral ischemia/reperfusion injury.

Hydrogen sulfide is the third gasotransmitter. In this study, the data indicate that NaHS attenuates cerebral ischemia/reperfusion injury induced by MCAO in rats and protects PC12 cells against OGD/R‐induced cellular injury. During the preparation of this article, Shui *et al*. 19 reported that H_2_S attenuates cerebral ischemia/reperfusion injury by inhibiting autophagy in mice. In combination with that work, the present study provides solid evidence that H_2_S is a protective factor against cerebral ischemia/reperfusion injury. In addition to the animal study, we also investigated the effects of H_2_S on neuronal cell injury induced by OGD/R, the *in vitro* model of ischemia/reperfusion, while Shui *et al*. did not test the protective effect of H_2_S *in vitro*. Our findings give more detail, demonstrating that H_2_S attenuates cerebral ischemia/reperfusion injury by inhibiting overactivated autophagy in rats.

Aside from autophagy, other mechanisms may also be involved in the protective effect of H_2_S against cerebral ischemia/reperfusion injury. Preconditioning with H_2_S inhalation protects against cerebral ischemia/reperfusion injury by inducing HSP70 through the PI3K/Akt/Nrf2 pathway 29. In an *in vitro* study, it was found that the protective effects of H_2_S are mediated by thiosulfate, which exerts antiapoptotic effects via persulfidation of caspase‐3 30. Furthermore, NaHS protects the brain against ischemia/reperfusion injury by inhibiting oxidative stress and apoptosis 31. In the present study, we demonstrate a new mechanism in which H_2_S attenuates cerebral ischemia/reperfusion injury by inhibiting overactivated autophagy.

One limitation of this study is that the endogenous H_2_S was not detected. The CBS, CSE, and 3‐mercaptopyruvate sulfur transferase/cysteine aminotransferase (3‐MST/CAT) pathways are involved in the generation of endogenous H_2_S. CBS, 3‐MST/CAT, and 3‐MST/D‐amino acid oxidase (DAO) can be found in the brain 31. In chronic kidney disease, the renal and hepatic H_2_S‐producing enzymes and capacity are downregulated 32. Given the potent antioxidant, anti‐inflammatory, and cytoprotective properties of H_2_S, its downregulation may contribute to the progression of cerebral ischemia/reperfusion injury. In further research, the expression and capacity of H_2_S‐producing enzymes will be investigated.

In conclusion, this study demonstrates that H_2_S attenuates cerebral ischemia/reperfusion injury both *in vivo* and *in vitro*. The mechanism of the protective effect of H_2_S is partially associated with suppression of overactivated autophagy.

## Author contributions

LXW and WWJ conceived and designed the experiments. WWJ and LHD performed the experiments. WWJ, BSH, and YH analyzed the data. LXW and WWJ contributed reagents/materials/analysis tools. LXW and WWJ wrote the manuscript. LXW and YH critically reviewed the manuscript.
